# Highly Conductive Paths in Diamond and their Application in High Pressure Measurements

**DOI:** 10.1021/acsami.4c12113

**Published:** 2024-10-17

**Authors:** Mateusz Gramala, Andrzej Sikora, Aleksandra Chudzyńska, Łukasz Gelczuk, Filip Dybała, Paweł Modrzyński, Robert Kudrawiec

**Affiliations:** † Nanores Sp. z o.o. Sp. k., Bierutowska 57-59, Wrocław 53-317, Poland; ‡ Department of Semiconductor Materials Engineering, Wrocław University of Science and Technology, Wybrzeże Wyspiańskiego 27, Wrocław 50-370, Poland; § Department of Nanometrology, Faculty of Electronics, Photonics and Microsystems, Wrocław University of Science and Technology, Janiszewskiego 11-17, Wrocław 50-372, Poland; ∥ Department of Optical Spectroscopy, W. Trzebiatowski Institute of Low Temperature and Structural Research of the Polish Academy of Sciences, Okólna 2, Wroclaw 50-422, Poland

**Keywords:** diamonds, conductive paths, focus ion beam, high hydrostatic pressure, diamond anvil cells

## Abstract

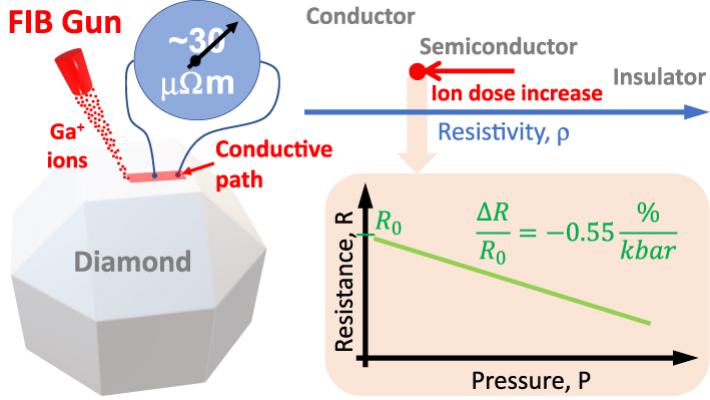

Diamonds possess exceptional properties such as high mechanical strength, thermal conductivity, and electrical resistance, making them suitable for various applications, including high-power electronics and optoelectronics. However, fabricating conductive structures in diamond remains a significant technological challenge. In this publication, we present a controlled process for fabricating precise amorphous conductive paths in a monocrystalline diamond using a focused ion beam (FIB) technique. The resistivity and thickness of the fabricated structures were determined, and their morphology was carefully characterized. The results showed that the optimal charge dose for achieving the lowest resistance was found to be 10^17^ ions/cm^2^. The fabricated structures exhibited amorphous morphology. Elemental analysis confirmed the presence of gallium ions in the modified material. The resistivity of the conductive paths was determined to be 30 μΩm, which is remarkably low compared to previous studies and only 1–2 orders of magnitude higher than in metals. Additionally, it is shown that this technology has potential applications in high-pressure chambers and the development of high-pressure sensors within diamond anvils. Overall, this study provides valuable insights into fabricating conductive structures on diamonds using FIB and opens up possibilities for diamond electronics.

## Introduction

Diamonds withstand extreme conditions, because of a number of excellent properties such as crystallographic structure of high symmetry, great mechanical strength, high electrical resistance and very good thermal conductivity.^1,2^ The diamond crystal has a wide band gap (5.5 eV), which makes it optically transparent in a wide spectral range. This material is considered to be ideal for use in high power and high temperatures electronics. It does not absorb infrared radiation, making it an ideal material for advanced optoelectronics applications.^3−5^ Until now, infrared sensors, high power laser elements and Raman lasers have been fabricated using diamonds.^6−8^ The excellent tribological properties of diamond can be exploited to enhance the wear resistance of microelectromechanical systems. In addition, the high chemical inertness as well as biocompatibility and excellent thermal conductivity make diamond an ideal material for fabricating biosensors or lab-on-a-chip solutions.^9^ The excellent mechanical parameters of diamond allow its use in high-pressure chambers, called diamond anvil cells (DAC), designed to obtain high hydrostatic pressure up to 100 GPa^10−12^ used for structural, optical and electrical studies of solids.

The potential use of diamond in electronics creates a need for technology for producing conductive structures on diamond.^13−15^ The development of a controlled process for producing precise paths in diamonds with satisfactory electrical parameters would enable easy and repeatable use in diamond electronics. However, this goal is still a technological challenge. One of the possibilities for producing the mentioned structures is the use of a focused ion beam.

Focused ion beam (FIB) is a technique used particularly in the semiconductor or materials science industry for sample analysis, deposition and ablation of materials.^10−12^ The use of FIB has become very popular in fabricating structures at the micro- and nanoscale, e.g., photonic crystals,^13−15^ the structure for optical waveguide^16−18^ or modification of diamond tip of atomic force microscopy (AFM) cantilever.^19,20^ The technology holds great promise for use in fabricating components for quantum computers with defects in diamond.^21−24^ The FIB is also commonly used to fabricate samples called lamellas, dedicated to transmission electron microscopy (TEM) analysis. This is one of the few methods of making samples transparent to the electron beam that is currently widely used.

Modification of a diamond with a FIB leads to local surface amorphization of the diamond layer as a result of ion implantation.^25−27^ This process significantly affects the density of the material, its crystallographic structure, as well as its optical and electrical properties.^28−31^ The thickness of the amorphous layer formed during surface modification with FIB was analyzed by Tong and Luo.^32^ Stages of the diamond structure changes depending on the dose of charge delivered to the surface and energy of the beam were studied by McKenzie et al.^33^ The diamond conductivity changes were also studied in connection with temperature sensor’s development.^34,35^ Structures made using FIB have so far been investigated by Pea et al.^36^ Authors described the measurements of thickness of manufactured structures and determined the resistivity of the diamond surface modified by Ga^+^ ions. They also presented current–voltage (*I*–*V*) characteristics and conductive AFM measurements of conductive paths. These structures show that the electrical discharge of the diamond sample is problematic, which negatively affects the process repeatability.

Previous works show that it has not yet been possible to develop a repetitive technology for fabricating structures with electrical parameters enabling the production of diamond electronics. None of the cited works clearly defines the mechanism of conductivity occurring in a diamond modified with the use of FIB. The determination of resistivity of structures produced using FIB in monocrystalline diamond will allow to design complex conductive structures in a controlled way, according to predefined parameters. This will have a significant impact on the development of the technology of manufacturing electrical systems in diamond structures. Such devices could be applied as sensors in high-pressure chambers, e.g., beforementioned DAC or other applications.

Currently, pressure measurements in DAC are performed using spectrometers that measure photoluminescence from ruby spheres placed inside the DAC. The pressure inside the DAC is determined based on the emission shift from these spheres.^37^ A much simpler and cheaper solution could be to measure pressure in the DAC using electrical methods, as is the case in Unipress anvils,^38,39^ but conductive paths in diamond are needed for this purpose. Such paths are also very desirable in electrical measurements performed in DAC because the previous solutions are not durable and cause many measurement problems. Therefore, the development of technology for producing repeatable and durable conductive paths in diamond is very interesting for high-pressure measurements with DAC.

In this paper, we present and discuss the technology to fabricate amorphous conductive pathways on a monocrystalline diamond substrate obtained by chemical vapor deposition (CVD) using a Ga^+^ FIB. We measured the resistance and thickness of these structures, determined their morphology, and determined the resistivity of the material formed by modifying the diamond structure with the Ga^+^ FIB. Next we demonstrated the application of the conductive paths in high pressure sensing.

## Experimental Section

Commercially available 500 μm thick CVD bulk-like diamond substrates from GaNova Electronic Information Co., Ltd. were used for the experiments. According to producer information the nitrogen concentration in these substrates is below 50 ppm, their thermal conductivity is about 2000 W/(m K) and the resistivity is >10^8^ Ωm.

Ion beam modifications of diamond substrates and their analysis by electron microscopy were performed using a Helios Nanolab 600i dual beam microscope equipped with a liquid metal ion source of Ga^+^. The beam accelerating voltage range is 2–30 kV. In addition, the device is equipped with a field electron emitter that allows imaging of the fabricated structures. Due to the working characteristics of FIB, all processes were performed under vacuum conditions. A well-known problem in the interaction of an ion beam with insulating samples such as diamond is the accumulation of electric charge. In our case, in order to remove this charge from the sample, first about 15 nm of amorphous carbon was deposited on the diamond substrate. Then, during the process of generating conductive paths, the EasyLift micromanipulator, which is grounded and which allows for effective discharge of the sample during the operation of the ion beam, was applied to the substrate surface inside the microscope. Once the conductive path formation is complete, the amorphous carbon layer is removed from the sample using a plasma cleaner in a process lasting 15 min. Samples for TEM were also fabricated from the structures and atomic scale imaging was performed using an FEI Titan 80–300 TEM. An electron beam of 300 keV energy was used for analysis in TEM.

In order to perform morphological analysis of the sample using transmission microscopy, it is necessary to prepare a lamella less than 100 nm thick, that is transparent to the high-energy electron beam. The sample was made using a FIB. First, the region of interest (conductive structure on diamond) was chosen, and a protective layer of platinum was deposited on it using focused electron beam induced deposition and then using FIB induced deposition technologies. The layer is designed to protect the surface of the sample from damage that could be caused by the ion beam while removing material around the selected area. Material removal around the area of interest was then performed using FIB. Using a micromanipulator, the lift-out process was performed, and the cut piece of the sample was mounted in a dedicated TEM microscope holder. Once the sample was properly mounted in the holder, the lamella was polished with a FIB. The lamella is polished on both sides with the ion beam to reduce its thickness and expose the microstructure. The polishing is carried out until the sample is transparent to a 5 kV electron beam, allowing high-resolution imaging of the structure using TEM.

MiBot manipulators from Imina with a positioning accuracy of 50 nm and an electrical meter 34450A from Agilent were used for electrical characterization of the paths. Since the measured resistances of the paths are large, a two-point resistance measurement was used.

To determine the changes in the diamond topology after the modifications made with FIB, measurements were made using a Nanosurf FLEX-Axiom AFM microscope. AFM characterization was performed in Taping mode, using Nanosurf’s dedicated AFM cantilevers. Elemental analysis of the structures was performed using a Bruker Xflash 630 EDS detector.

## Results and Discussion

**Figure 1:**
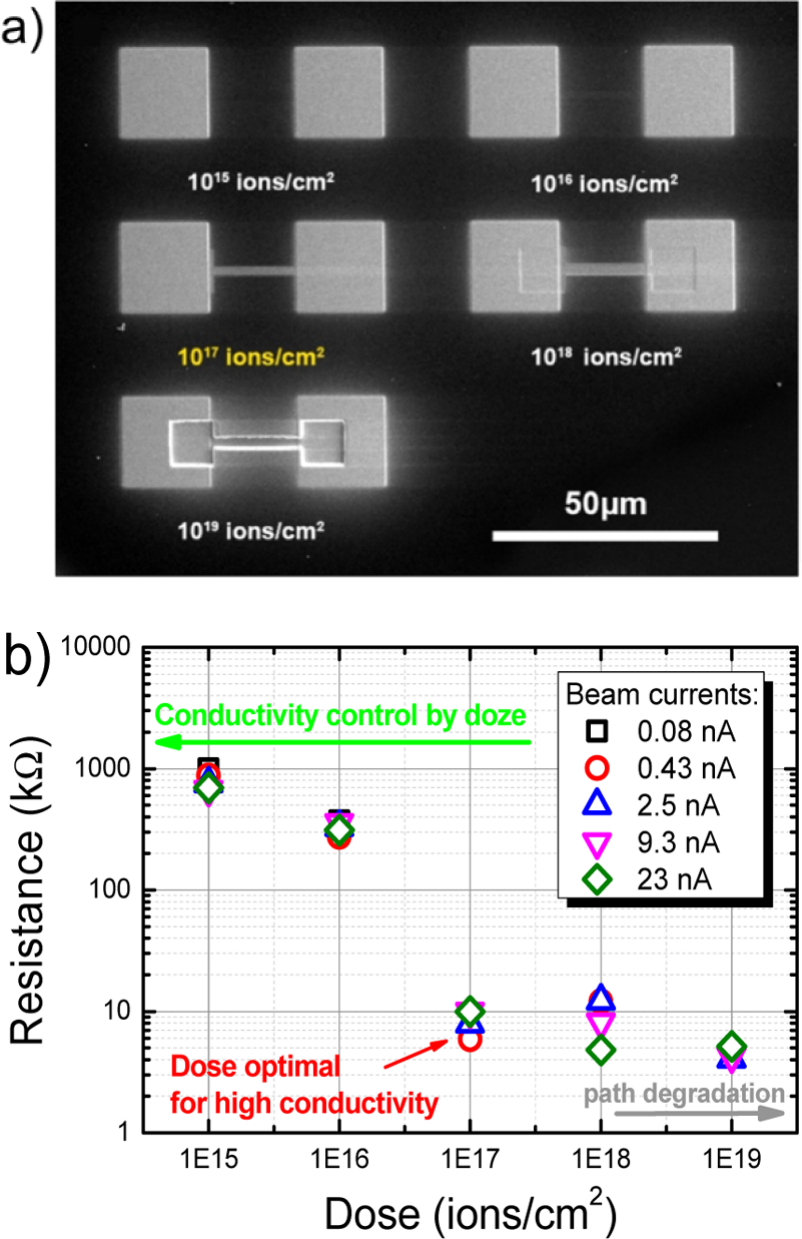
(a) SEM images of conductive paths obtained for different Ga^+^ dose at the current of 0.43 nA. (b) Resistance of conductive paths obtained for different Ga^+^ dose and current.

First, conductive paths were fabricated, where the charge dose delivered to the substrate was a variable parameter (Figure 1). The Ga^+^ dose was varied from 10^15^ to 10^19^ ions/cm^2^ in 1 order of magnitude increments. In addition, structures were made using beam currents changing in the range of 0.08–23 nA. The current values depended on the apertures used in the FIB microscope. In this way, five sets of samples with different ion doses were produced at five different currents to determine the optimal conditions for producing conductive paths.

In order to ensure stable electrical contact between the probe and the structure, after the implantation and amorphization processes, an organometallic precursor was introduced into the microscope chamber on the sample surface, using an appropriate gas injection system. Then, using FIB induced deposition technology, platinum contact fields were sputtered. The fabricated structures are shown in Figure 1a.

**Figure 2:**
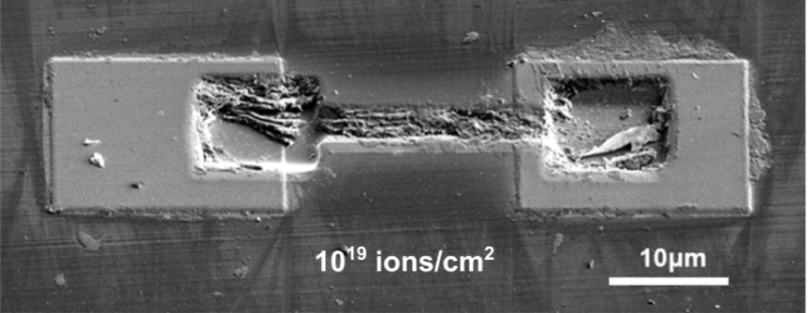
SEM image of conductive paths obtained at too high Ga^+^ dose and at the current of 0.43 nA.

The I–V characteristics were measured for the obtained paths and their resistances were determined based on these measurements. We observed that by changing the ion dosage from 10^15^ to 10^19^ ions/cm^2^, the resistance changes at least 2 orders of magnitude from ∼1000 kΩ to a few kΩ, while change in current does not change the resistance significantly, see Figure 1b. In addition, it is an important fact to make paths with the best electrical parameters before material removal begins, due to the gallium ion influence with the diamond surface, see the unwonted situation in Figure 2. Therefore, the optimal charge dose, for which the path resistance reaches the lowest value, was determined to be at 10^17^ ions/cm^2^, see Figure 1b. Further increase in Ga^+^ dose does not significantly change the resistance, but the material from the conductive path is removed, see Figure 2. In general, this observation is consistent with previous studies of ion interaction with diamond,^40^ in which the formation of damage layers near the surface was observed. However, possible damage can be effectively eliminated in this technology by using a low ion dose. In our case, the dose of 10^17^ ions/cm^2^ and lower is the dose at which no surface damage is observed, as evidenced by SEM images and as shown later in TEM analysis for the conductive paths obtained at a dose of 10^17^ ions/cm^2^. Hence, it can also be concluded that the mechanical stability of the sample as a whole is not significantly affected at such low doses.

It was also determined that changing the beam current does not affect the electrical material parameters of the path, but it does affect the duration of the process and the resolution of the fabricated structures. Hence, we conclude that lower currents are more recommended for producing conductive paths.

**Figure 3:**
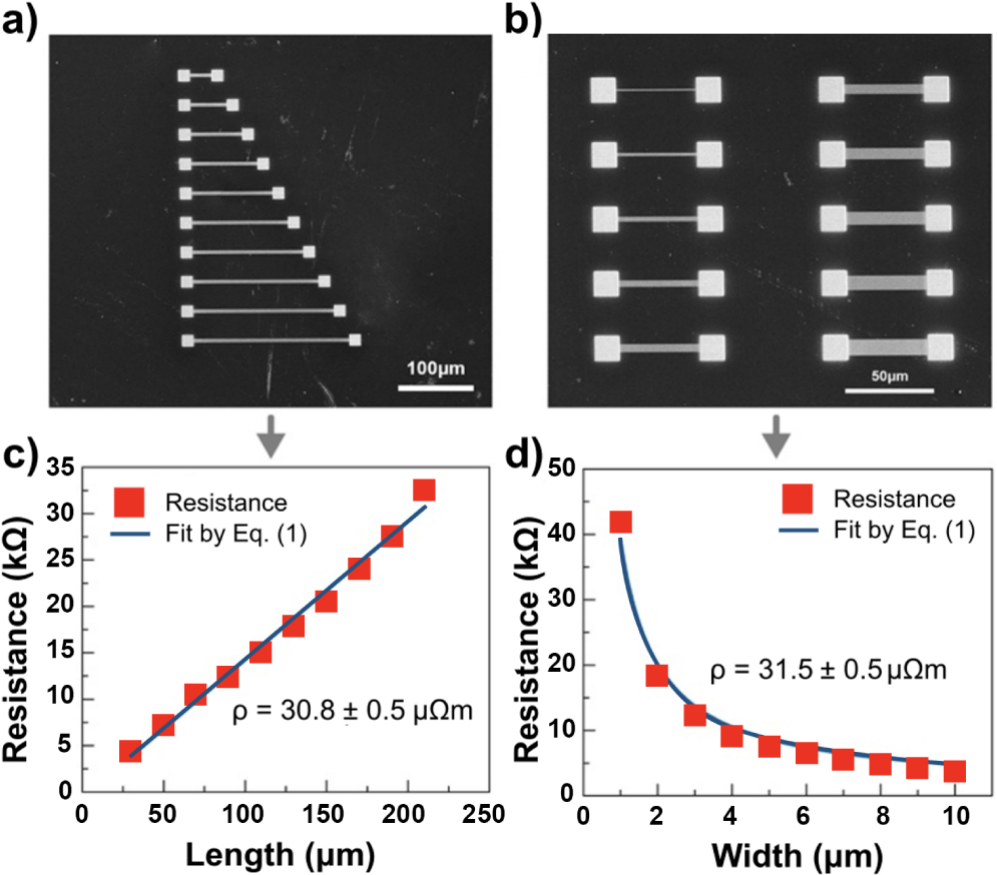
Conductive structures on diamond made for the purpose of determining the resistivity of the material fabricated by the interference of a focused gallium ion beam on the diamond. Ten structures with fixed width and variable length (a) and ten structures with fixed length and variable path width (b). Resistance measured for the set of paths with different length (c) and different width (d).

The next step is to determine the resistivity of the paths with the lowest resistance. The knowledge of this material parameter is crucial for the design of conductive structures with assumed electrical parameters. To determine the resistivity (ρ) of the material, the basic physical formula given in eq1 was used:
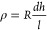
1where *R*, *d*, *h*, and *l* is the resistance, width, thickness and length of the pathways, respectively. Two sets of samples were prepared to determine the resistivity. One series with the same width and different length and another series with the same length and different width, see Figure 3a,b, respectively.

For the resistivity calculations, according to eq 1, it was necessary to determine the thickness of the layer formed due to the interaction of a focused gallium ion beam into the diamond structure. For this purpose, proper lamellas were fabricated and TEM studies were performed.

**Figure 4:**
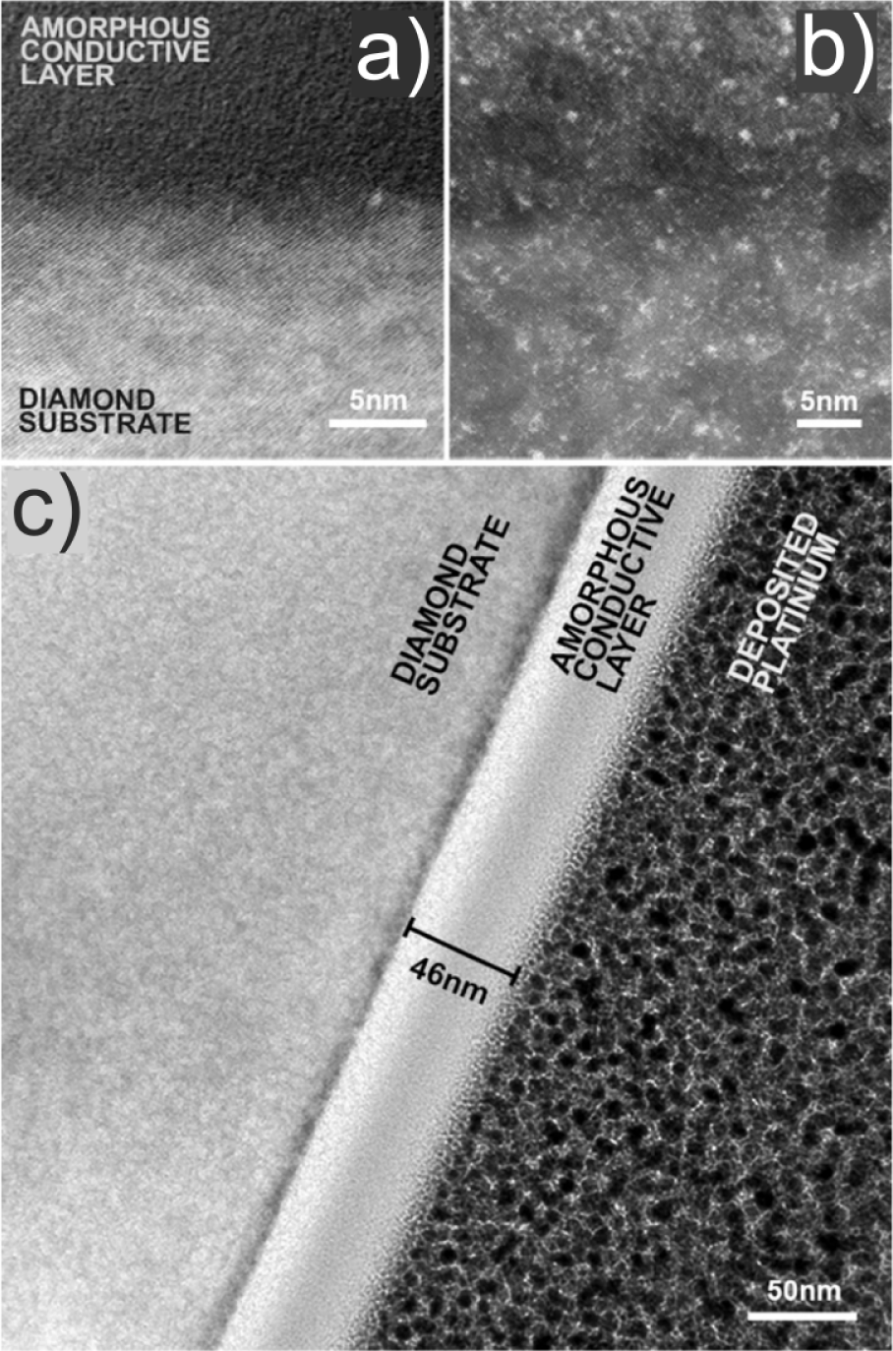
TEM images of an exemplary conductive path in diamond, formed by modification with FIB. (a) The morphology of the interface between the diamond and the conductive path. (b) The image from the HAADF detector. (c) The conductive path shown in scale, allowing determination of its thickness.

TEM images clearly show that the resulting layer is an amorphous material, see Figure 4. The interface between the conductive path and diamond is well-defined and therefore the thickness of the conductive path can be accurately determined. For the conductive path, obtained at the optimal FIB conditions (10^17^ ions/cm^2^), its thickness is equal to 46 ± 1 nm, according to Figure 4c. Figure 4b was obtained from the high angle annular dark field (HAADF) detector and shows the chemical contrast of the material. The bright spots, visible in the image, are atoms of elements heavier than carbon and therefore they are attributed to gallium atoms.

**Figure 5:**
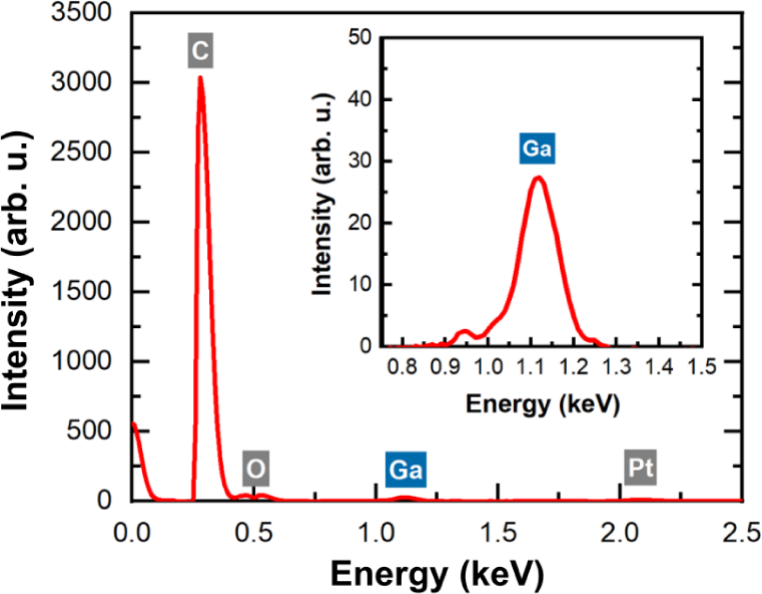
EDS spectra of a conductive structure fabricated in diamond using a focused gallium ion beam.

The presence of gallium in the material was confirmed by elemental analysis using energy-dispersive X-ray spectroscopy (EDS). An electron beam with the energy of 5 kV and a current of 3.2 nA was used as an excitation for the material, to generate characteristic X-ray radiation. Figure 5 shows the EDS spectrum of one of the studied samples, with the strongest peak at 0.28 keV, associated with carbon (C) atoms. In addition to the C peak, a peak at ∼1.1 eV is observed, see inset in Figure 5. This peak is 100 times weaker and it is attributed to gallium (Ga) atoms, which are present in the conductive paths. Ga peak was not observed for diamond substrate outside the conductive path area. In this case only C and oxygen (O) peaks were observed. The presence of oxygen on the diamond surface is rather typical for samples, which were stored in ambient conditions and which surface was not treated specially before EDS measurements. It is possible that gallium oxide is also formed in conductive paths, but we do not have strong evidence for this.

**Figure 6:**
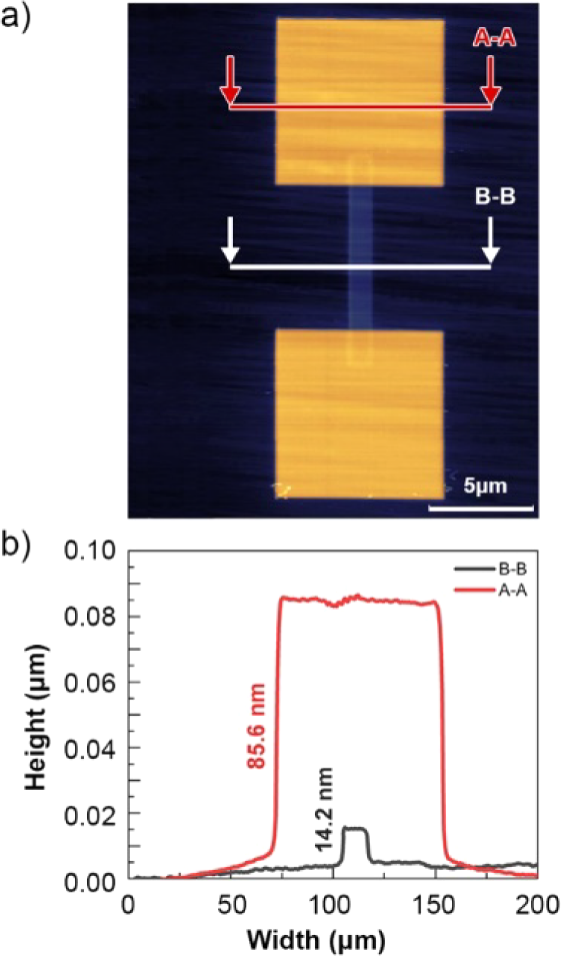
(a) AFM analysis of the fabricated structures, showing the convexity of the conductive path (the orange areas are platinum contacts). (b) The A–A and B–B profile showing the height of the path and platinum contacts, respectively.

Further studies of the topography of conductive paths were performed using AFM. Figure 6 shows AFM image of the conductive path with Pt pads together with profiles across the Pt pad (A-A) and the conductive path (B–B) for sample obtained at the dose of 10^17^ ions/cm^2^. The AFM analysis clearly shows that the structures become convex and increase in volume by approximately 30%, i.e., ∼ 14 nm of the 40 nm conductive path is above the diamond surface. This is caused by the amorphization of the diamond, through its implantation with gallium ions.

Knowing the sizes of the conductive paths (*d*, *h*, and *l*), the resistance measurement results for the series of samples with different lengths (Figure 3a) and different widths (Figure 3b) can be fitted by eq 1with ρ as a free parameter. The obtained resistivities are equal to 30.8 ± 0.5 and 31.5 ± 0.5 μΩm for the set of samples with different lengths and widths, respectively, and are almost the same within the experimental accuracy. These results are very consistent with those reported in ref (34); however it should be noted that the authors in ref (34) studied very narrow paths (∼20 nm) and observed the change of conductivity of these paths with their length, which suggests additional effects in such narrow paths. In our case the resistivity (conductivity) does not depend on the path sizes (see that the deviations from the theoretical curves in Figure 3c,d are within the measurement accuracy) and therefore the path resistance can be predictably determined by the geometrical size of the paths which is an obvious conclusion considering Ohm’s law.

**Figure 7:**
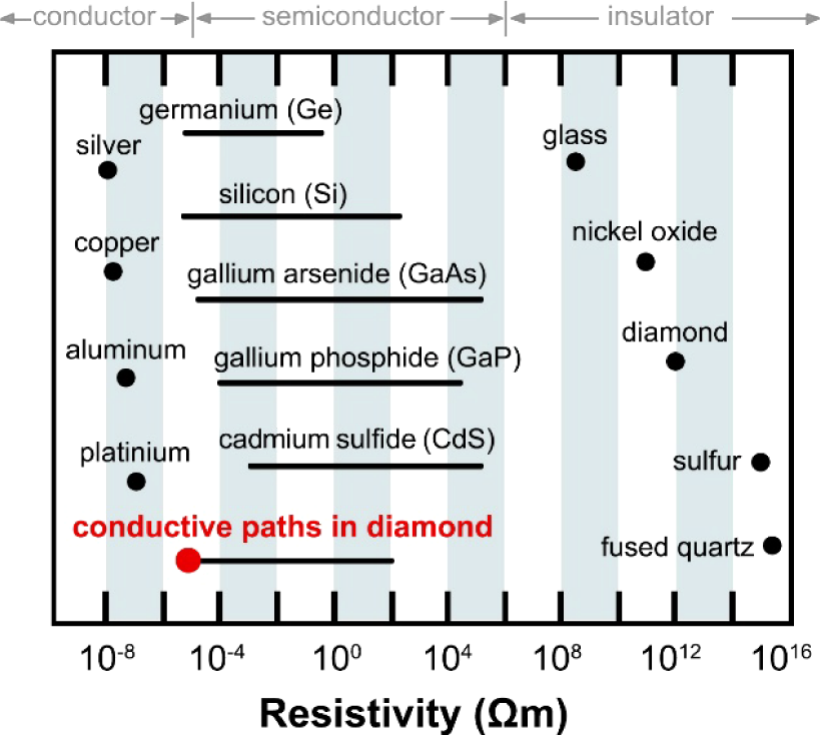
Resistivity of selected insulating materials, semiconductors and metals along with the resistivity of conductive paths in diamond. The red dot indicates the lowest resistivity obtained in this work. The horizontal line starting from the red dot is an estimate of the available resistivity of the conductive paths in the diamond, which is based on the results presented in this paper (the results for a lower ion dose are shown in Figure 1) and refs (34 and 36). The control of the conductive path resistivity in this case can be achieved by the ion dose, which can vary from 10^17^ to 10^12^ ions/cm^2^ or even lower. In general, the limit of the highest resistivity in this case is defined by the resistivity of the diamond before ion treatment, which for diamonds studied in this paper is 10^8^ Ωm.

Figure 7 shows how the achieved resistivities of conductive paths in diamonds compare to the resistivities of other insulating materials, semiconductors and metals. First, it should be emphasized that the resistivity of ∼31 μΩm is remarkably low compared to previous studies,^36^ comparable to ref (34) and only 2 orders of magnitude higher than the resistivity of metals. Second, this technology of producing conductive paths, gives us the ability to control their resistance by at least 3 orders of magnitude, which can be used to design electronic circuits with such paths.

As it is known and shown in Figure 7, the resistivity of metals cannot be changed by orders of magnitude, while the resistivity of semiconductors can vary by several orders of magnitude. Therefore, our conductive paths can be compared to semiconductors in terms of the possibility of changing resistivity. However, it should be underlined that conduction in this type of paths has a different nature than conduction in semiconductors, where the conductivity is controlled mainly by changing the concentration of electrons in the conduction band (*n*-type semiconductor) or holes in the valence band (*p*-type semiconductor).

In the studied paths, unlike metals and semiconductors, conductivity is not well described and can be very complex. In amorphous areas the resistivity is typical as for amorphous carbon where it ranges from 1.5 to 4.5 × 10^–5^ Ωm. Our TEM analysis of the paths obtained at a dose of 10^17^ ions/cm^2^ shows that these paths are amorphous and quite homogeneous, see Figure 4. The resistivity of this path (∼3 × 10^–5^ Ωm) is in the range typical for amorphous carbon. Therefore, in this case, it can be assumed that the conduction mechanism is similar to that in amorphous carbon. We can speculate that in paths with higher resistivity (those obtained with a smaller dose of ions) the contribution of the amorphous part to conductivity is smaller and the contribution of the nonamorphous part with an insulating character increases, increasing the resistance of the entire path. According to our structural studies (TEM and EDS), there may be gallium-rich regions in the conduction paths, visible in Figure 4b as nanometer-sized bright spots in this case. This material object cannot be neglected in the conduction mechanism of these paths for the reason that it is conductive itself and, moreover, it can be a source of electrons for its surroundings. This means that we can have at least three material motifs in the considered paths: amorphous (conducting amorphous carbon), nonamorphous (nonconducting diamond) and metallic (conducting gallium). These motifs can have different scales and can be considered on different scales.

In the nanoscopic image, electron hopping inside an insulating area or between a conducting and insulating area can be one of the mechanisms responsible for the flow of current in the studied paths. In the microscopic image, these areas of different conductivity are connected in series and parallel and such a system can be viewed as an electrical circuit of interconnected resistors between which there is a flow of electrons. It is, therefore, a heterogeneous system in which heterogeneity (contribution of amorphous, nonamorphous, gallium-rich areas, their size, etc.) controls the electronic conductivity. This heterogeneity can be controlled using the ion beam and annealing after the beam treatment process. However, after creating a conductive path, this inhomogeneity remains and the conductivity of the path does not change.

This means that the conduction mechanism in FIB paths in diamond cannot be controlled like the conduction in semiconductors, especially in devices such as transistors where the carrier concentration in the conduction channel is controlled by an external voltage. Nevertheless, it can be expected that external factors may influence the resistance of such paths. One of such factors is the hydrostatic pressure and this factor has been analyzed for the use of this type of paths in high pressure sensors as it is shown in the next section.

## Applications

We believe that the described technology for producing conductive paths with a record low resistivity, has great potential in diamond-based optoelectronics. We mean both high-power transistors, where electrical contacts are necessary, and single photon sources based on color centers in diamond. However, it is unlikely to be possible to demonstrate the usefulness of our paths in this type of devices at present, because such devices are at an early stage of development, but it is possible to demonstrate the use of conductive path technology in high pressure sensors.

**Figure 8:**
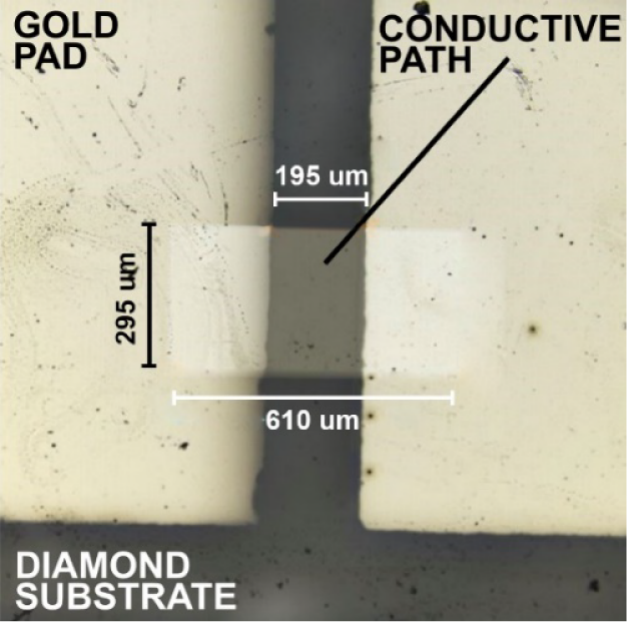
Microscope photography of the conductive path with gold pads made on the diamond substrate.

**Figure 9:**
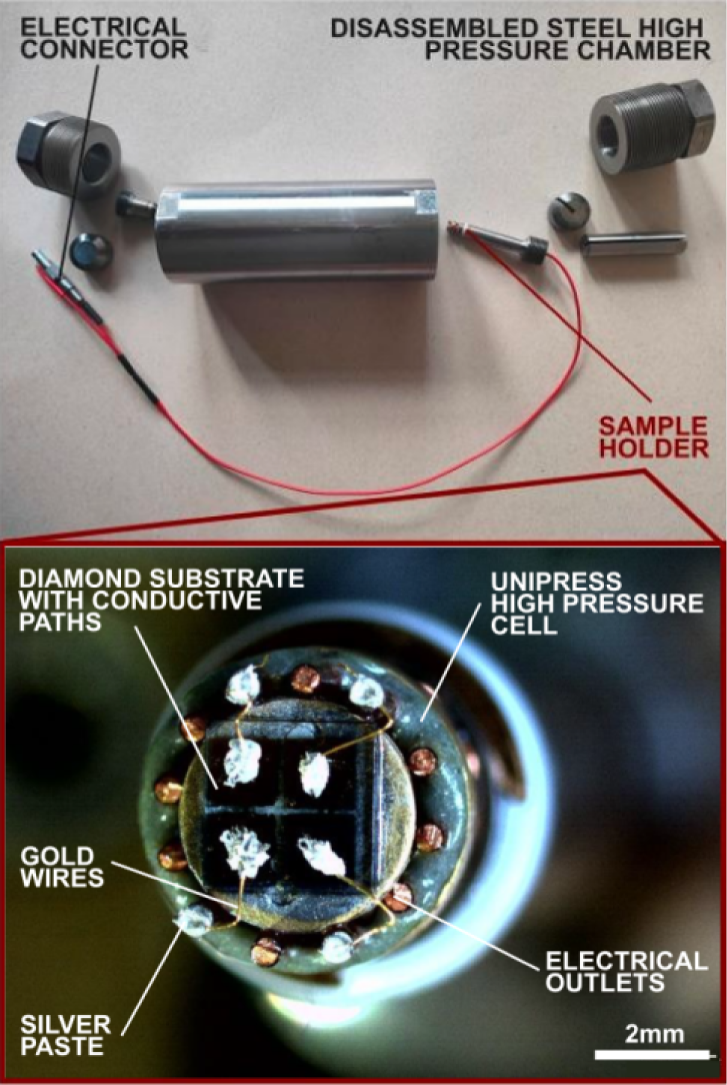
Steel high-pressure chamber with mounted diamond substrate, electrically connected to the connector dedicated for electrical analysis with pressure.

Measuring pressure inside a DAC designed for measurements under high hydrostatic pressures is not that easy. At present, it requires a sophisticated optical measurement system to measure the photoluminescence of ruby spheres.^37^ One application of the technology described in this work could be to produce of high-pressure sensors, directly within the volume of the DAC. As part of the experiment, conductive paths with defined shapes were made on the diamond substrate. In the next step, gold contact fields were deposited onto the diamond substrate, to allow easy electrical connection to the path, see Figure 8. The substrate made in this way was placed in an Unipress high-pressure chamber^38,39^ and electrical contacts were brought outside the chamber, see Figure 9.

**Figure 10:**
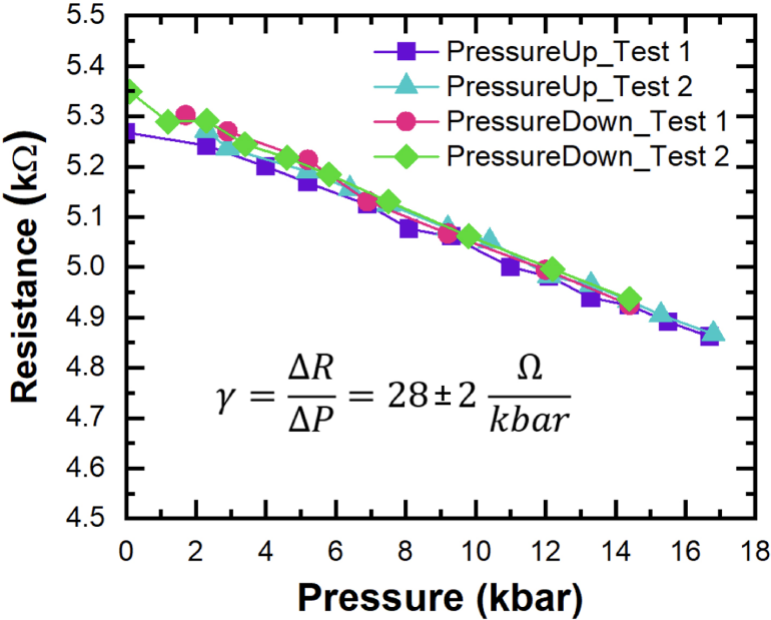
Resistance of a conductive path in diamond as a function of applied hydrostatic pressure. This slope γ gives a relative change in resistance of ∼0.55%/kbar.

In the experiment, the hydrostatic pressure in the chamber was increased up to ∼18 kbar. At the same time, the resistance of the path was measured and its change as a function of hydrostatic pressure was observed. The measurement was carried out twice during the increase and decrease of pressure, and the results of both tests were shown in Figure 10. The obtained results show that conductive paths made in diamond using FIB technology respond linearly and repeatably to the changes in the hydrostatic pressure applied to them, see Figure 10. The obtained coefficient of change in resistance under the influence of pressure is negative and equals to −28 ± 2 Ω/kbar. In the case of InSb, which is routinely used for pressure measurements in Unipress chambers and was used in this case, the resistance increases with increasing pressure and this increase is nonlinear.^41^ This is related to the opening of the energy gap in InSb under the hydrostatic pressure and the associated decrease in carrier concentration. The mechanism of resistivity changes of the conductive paths in diamond is different than that observed in InSb and therefore in this pressure regime the opposite behavior of resistance is observed for conductive paths and InSb. A very important conclusion from the measurements presented in Figure 10 is that the resistance values after several such pressure cycles are preserved, i.e., after removing the high pressure these resistances at atmospheric pressure are the same within the measurement accuracy as before the experiments. Furthermore, the resistances of such paths are preserved over time and so far we have not observed anything indicating a temporary degradation of such paths.

The decrease in resistance of the conductive path with an increase in hydrostatic pressure can be explained by the enhancement of the electron hopping process in the FIB treated diamond due to the reduction of the distance between the hopping centers as well as between the conducting areas. Since the obtained pressure characteristics are linear, the path made using FIB technology is a very suitable medium for pressure sensors. Additionally, FIB paths can be electrical connections to the sensor active area in the DACs.

The main factor, that distinguishes the conductive paths in diamond presented in this work from the standard metal paths on diamond is their high durability and resistance to high temperatures. This durability is due to the fact that these paths are inside the diamond and not on its surface. Only 30% of the path protrudes above the surface of the diamond, but this is one block of material, unlike the metal paths, which are on the surface of the diamond and attach to the diamond by cohesive forces. Due to the different thermal expansions of the diamond and metal, the metal paths may detach from the diamond. This is not the case with paths made with FIB technology. This feature gives an advantage over metal paths on diamond, which are much less mechanically durable and degrade more easily, especially at higher temperatures and under the influence of friction. Therefore, making conductive paths in diamond using FIB technology also has great prospects for use in DAC with electrical access for electrical measurements, where metal paths are currently used and where due to the friction between the diamond and the gasket, such metal paths degrade very quickly and are often disposable.

In the case of the Unipress chamber, the pressure sensor produced in FIB technology on diamond has sufficient sensitivity to replace the InSb sensor. Additionally, in the applied pressure range it has the linear response, which is also its great advantage. Finally, it is worth noting and emphasizing that the DAC with electrical access made in FIB technology, combined with the sensing part inside the measurement chamber also made in FIB technology, will be a system that does not require a spectroscopic PL equipment for pressure measurements, just like the Unipress chamber. Here it is important to emphasize that achieving permanent electrical connections in DAC is still a big challenge in high-pressure electrical measurements. We are convinced that this challenge can be met within the technology discussed in this article and our work on such paths in DAC is the next step in this research and will be reported in the future.

In addition, the next step in the development of conductive diamond path technology may be mastering the repeatable annealing of these paths. So far, it has been reported that annealing can improve conductivity (reduce resistivity) by 2 orders of magnitude.^34^ Our studies confirm this, but also show that repeatability of results may be a big challenge here because the resistivity of annealed paths is very dependent on the annealing temperature and time.

## Conclusions

In conclusion, our study demonstrates the successful fabrication and characterization of conductive paths in monocrystalline diamond using the FIB technique. We showed how to change the resistivity of conductive paths in diamond from 10^12^ to 10^–5^ Ωm. The resistivity of the material obtained at the optimal ion dose of 10^17^ ions/cm^2^ was determined to be ∼30 μΩm, which is remarkably low compared to previous studies and only 2 orders of magnitude higher than in metals. A conduction mechanism in these paths was proposed and the pressure-induced resistivity decrease was explained within this mechanism. For the Unipress high-pressure chamber, a pressure sensor based on the conductive path in diamond made by FIB was fabricated and used to measure hydrostatic pressure. Additionally, the advantages of using FIB technology to produce conductive paths in the DAC were discussed.
